# Effects of Short-Term Quercetin Supplementation on Urinary Nicotine Metabolism Biomarkers in Users of Conventional and Alternative Nicotine Products: A Repeated-Measures Study

**DOI:** 10.3390/toxics14070591

**Published:** 2026-07-05

**Authors:** Antonia Zecic, Ana Vucak, Ajka Pribisalic, Nada Bilopavlovic, Franko Burcul, Nina Kalajzic, Sendi Kuret, Ana Batinic, Livia Sliskovic, Davorka Sutlovic

**Affiliations:** 1 Department of Natural and Biomedical Sciences, Faculty of Forensic Sciences, University of Split, 21000 Split, Croatia; azecic@forenzika.unist.hr (A.Z.); lsliskovic@forenzika.unist.hr (L.S.); 2 Department of Analytical and Environmental Chemistry, Faculty of Chemistry and Technology, University of Split, 21000 Split, Croatia; ana.vucak@ktf-split.hr (A.V.); franko@ktf-split.hr (F.B.); 3 Faculty of Health Sciences, University of Split, 21000 Split, Croatia; apribisalic@fzz.unist.hr (A.P.); nada.bilopavlovic@kbsplit.hr (N.B.); nkalajzic@fzz.unist.hr (N.K.); sendikuret@gmail.com (S.K.); 4 Department of Medical Laboratory Diagnostics, University Hospital of Split, 21000 Split, Croatia; 5 Pharmacy of Split-Dalmatia County, 21000 Split, Croatia; ana.batinic05@gmail.com; 6 Department of Applied Pharmacy, School of Medicine, University of Split, 21000 Split, Croatia

**Keywords:** nicotine metabolism, nicotine metabolite ratio (NMR), quercetin, urinary biomarkers, tobacco products, nicotine exposure

## Abstract

Nicotine is the main psychoactive component of tobacco and is considered to be the main substance responsible for the development of tobacco addiction. The main enzyme responsible for nicotine metabolism, CYP2A6, catalyzes the conversion of nicotine to cotinine and the subsequent metabolism of cotinine to *trans*-3′-hydroxycotinine. CYP2A6 activity is known to be modulated by various compounds, such as quercetin. This repeated-measures study examined the effects of short-term quercetin supplementation on urinary nicotine metabolism biomarkers in adult users of conventional and alternative nicotine products. Seventy-two participants completed a two-week study protocol involving first-morning urine collection at four time points: baseline, immediately after three days of quercetin supplementation (500 mg/day), seven days after supplementation, and ten days after supplementation. Urinary nicotine, cotinine, and *trans*-3′-hydroxycotinine concentrations were measured, and the nicotine metabolite ratio was calculated as *trans*-3′-hydroxycotinine/cotinine. Repeated-measures analysis of variance was used to evaluate biomarker changes over time according to sex, nicotine product type, and self-reported nicotine consumption intensity. Quercetin supplementation did not consistently alter nicotine metabolism biomarkers, while a descriptive increase in median urinary nicotine concentration after supplementation was observed in participants reporting lower daily nicotine consumption compared with other groups. These findings suggest that further studies are warranted to better clarify the effects of quercetin on nicotine metabolism across different levels of nicotine exposure.

## 1. Introduction

Tobacco use remains a major global public health threat, causing more than 7 million deaths annually, despite a gradual decline in tobacco consumption worldwide [1,2]. Nicotine is the primary addictive component of tobacco and the main driver of tobacco dependence [3,4]. It is predominantly metabolized by CYP2A6 to cotinine and subsequently to *trans*-3′-hydroxycotinine [5,6,7,8]. The nicotine metabolite ratio (NMR; *trans*-3′-hydroxycotinine/cotinine) is widely used as a phenotypic marker of CYP2A6 activity and nicotine metabolism [9,10,11]. Because CYP2A6 plays a central role in nicotine metabolism, compounds capable of modulating its activity, including naturally occurring dietary flavonoids such as quercetin, have attracted increasing research interest.

Urinary nicotine and its metabolites are commonly used biomarkers of tobacco exposure because urine provides a non-invasive and reliable matrix for their determination [12,13]. Nicotine metabolism exhibits substantial interindividual variability influenced by genetic, physiological, and environmental factors, including sex, age, ethnicity, diet, medications, and smoking status [5,12].

Alongside conventional cigarettes, the use of alternative nicotine products, such as e-cigarettes, heated tobacco products, and nicotine pouches, has increased considerably [2,14]. Although often perceived as less harmful than conventional tobacco products, the long-term health effects of these products remain insufficiently understood [15,16,17]. Furthermore, nicotine exposure may vary considerably according to product type and product characteristics. The growing diversity of nicotine products further complicates the assessment of nicotine exposure and nicotine metabolism across users [18,19,20].

In addition to variability in exposure, nicotine metabolism itself is a complex process that may be influenced by various external compounds. Research indicates that some antioxidant supplements, including quercetin, may affect nicotine metabolism by regulating enzymes associated with its biotransformation [21,22]. Quercetin is a flavonoid widely distributed in plant-based foods, including tea, wine, and various fruits and vegetables, with particularly high concentrations found in capers, dill, coriander, rocket, fennel, juniper berries, bee pollen, and okra [23]. In addition to its antioxidant and anti-inflammatory properties [23], quercetin has also been investigated for its potential to modulate drug-metabolizing enzymes, including CYP2A6, the primary enzyme responsible for nicotine metabolism. Studies have suggested that quercetin may inhibit CYP2A6 activity and reduce nicotine metabolism in vitro [24]. However, research indicates that the amount of quercetin typically obtained through dietary sources may be insufficient to meaningfully influence nicotine metabolism in vivo [25,26]. Although supplementation could theoretically provide higher exposure levels, studies conducted in humans have reported inconsistent findings, including increased CYP2A6 activity following quercetin supplementation [22]. Given the complexity of nicotine metabolism and the substantial interindividual variability arising from genetic, physiological, environmental, and behavioural factors, the direction and magnitude of the effects of controlled quercetin supplementation on nicotine metabolism remain uncertain. This uncertainty is particularly relevant in populations using different nicotine products, where substantial variability in nicotine exposure may further complicate the assessment of nicotine metabolism.

Therefore, the aim of this study was to investigate the effect of quercetin supplementation on urinary nicotine concentrations and nicotine metabolism biomarkers in users of conventional cigarettes and alternative nicotine products, including heated tobacco products and e-cigarettes. In addition, potential differences according to sex, product type, and nicotine consumption intensity were explored.

## 2. Materials and Methods

### 2.1. Participants, Recruitment, and Ethical Approval

**Figure 1:**
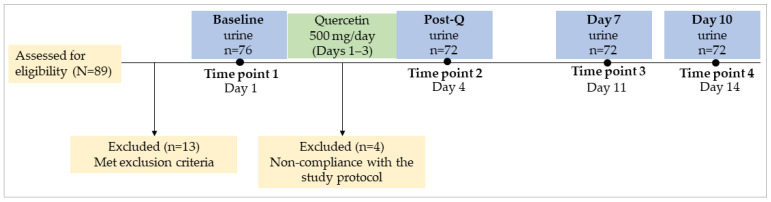
Participant flow diagram and study design with timeline of urine sample collection. Baseline, before quercetin supplementation (time point 1); Post-Q, after 3 days of daily quercetin supplementation (time point 2); Day 7, after 7 days of quercetin cessation (time point 3); Day 10, after 10 days of quercetin cessation (time point 4).

The study was conducted in Split, Croatia, at the University of Split, Faculty of Health Sciences, between October 2025 and February 2026. Participants were recruited through direct contact and voluntary participation. A total of 89 adult users of nicotine-containing products were assessed for eligibility, of whom 13 met the exclusion criteria. The remaining 76 participants were enrolled in the study, of whom 72 completed the study protocol and were included in the final analysis (Figure 1).

Eligible participants were individuals aged 18 years or older who regularly used nicotine-containing products, including conventional cigarettes, electronic cigarettes, heated tobacco products, and nicotine pouches.

Exclusion criteria were being under 18 years of age, presence of chronic diseases, pregnancy or breastfeeding, use of hormonal contraceptives, marijuana smoking, current use of quercetin supplements, use of medications known to affect nicotine metabolism, particularly through modulation of CYP2A6 activity, acute illness or infection at the time of urine sample collection, and inability or unwillingness to comply with the study protocol.

Prior to enrolment, all participants provided written informed consent and completed an interview. After enrolment, they received detailed instructions and all materials required for participation in the study. Ethical approval was obtained from the Ethics Committee of the University of Split, Faculty of Health Sciences (Class: 029-03/25-08/01; Registration No.: 2181-228-103/1-32; 23 April 2025).

### 2.2. Study Design and Procedure

Participants followed a two-week study protocol during which first-morning urine samples were collected at four time points. The first sample served as a baseline measurement (time point 1). Participants then received quercetin supplementation for three consecutive days, after which a second urine sample was collected (time point 2). Quercetin supplementation consisted of commercially available capsules (Healthy World, Be Healthy, Kranj, Slovenia) containing 500 mg of quercetin per capsule derived from Sophora japonica flower extract standardized to 98% quercetin. Participants consumed one capsule daily. The third urine sample was obtained seven days after quercetin supplementation (time point 3), followed by the collection of the fourth sample ten days after quercetin supplementation (time point 4). To ensure consistency across study time points, participants were instructed to take the supplements and collect urine samples at the same predetermined time throughout the study.

The study design is shown in Figure 1. Supplementation compliance was promoted through detailed written instructions, individualized supplementation schedules, and regular self-report assessments conducted at each sampling time point.

This study design enabled the assessment of within-subject changes in urinary concentrations of nicotine, cotinine, and *trans*-3′-hydroxycotinine following quercetin supplementation. Throughout the study period, participants were instructed to maintain their usual daily activities, dietary habits, and patterns of nicotine product use. No specific dietary restrictions were imposed.

### 2.3. Questionnaire and Data Collection

Participants completed a structured questionnaire designed to collect data on demographic characteristics, types of nicotine products used, and detailed information on product use, particularly for newer nicotine products. The questionnaire also included data on frequency and duration of use, as well as lifestyle factors such as dietary habits, coffee consumption, energy drink intake, and alcohol use. These data were used to characterize the study sample and to support subgroup analyses according to product type and intensity of nicotine consumption. Although participants were instructed to maintain their usual lifestyle and dietary habits throughout the study and dietary intake was not standardized, information on factors potentially affecting nicotine metabolism, including environmental tobacco smoke exposure, nicotine replacement therapy use, and consumption of quercetin-rich foods and other dietary factors known to modulate CYP enzyme activity, was collected at each sampling time point.

### 2.4. Classification of Nicotine Product Use

Participants were classified according to the type of nicotine product used into two groups: conventional cigarette smokers and users of alternative nicotine products, including heated tobacco products, electronic cigarettes, and nicotine pouches. Because only one participant reported nicotine pouch use, this product category was not considered separately.

Participants were additionally categorized according to the intensity of nicotine product use based on questionnaire data. Among conventional cigarette smokers, intensity was classified based on the number of cigarettes smoked per day as low (≤10 cigarettes/day), moderate (11–20 cigarettes/day), and high (>20 cigarettes/day). The same classification was applied to users of heated tobacco products, based on the number of tobacco sticks used per day.

For e-cigarette users, intensity of use was estimated based on theoretical daily nicotine intake, calculated as the product of the volume of e-liquid consumed per day (mL) and nicotine concentration (mg/mL). Nicotine concentrations reported as percentages were converted to mg/mL (e.g., 1% = 10 mg/mL). Based on the estimated daily nicotine intake, participants were categorized into low (<10 mg/day), moderate (10–20 mg/day), and high (>20 mg/day) exposure. Values exactly at the threshold of 20 mg/day were classified as moderate to ensure consistency. The participant using nicotine pouches was categorized according to the estimated daily nicotine intake.

Although different metrics were used to classify intensity across product groups, this approach was intended to reflect product-specific consumption patterns rather than to imply direct equivalence of systemic nicotine exposure.

### 2.5. Urine Sample Extraction

First-morning urine samples were collected at predefined time points according to the study protocol. After collection, urine samples were stored at −20 °C until analysis. Before sample preparation, urine samples were centrifuged for 5 min at 2000 rpm. Following centrifugation, 500 µL of the supernatant was separated for analyte extraction, while an additional 500 µL aliquot was reserved for creatinine determination. For the extraction procedure, 500 µL of urine was mixed with 10 µL of internal standard solution and 1 mL of acetonitrile. The mixture was vortex-mixed and subsequently centrifuged for 10 min. The resulting supernatant was then transferred for instrumental analysis. All urine samples were processed and analyzed in duplicate.

### 2.6. Reagents and Standards

Methanol and acetonitrile (LC-MS grade, CHROMASOLV™) were obtained from Honeywell (Charlotte, NC, USA) and were used for sample preparation and analysis. Analytical standards of S(−)-nicotine, cotinine, and *trans*-3′-hydroxycotinine (all 1.0 mg/mL in methanol), as well as the deuterated internal standard (±)-cotinine-*d3* (100 μg/mL in methanol), were obtained from Cerilliant (Round Rock, TX, USA).

### 2.7. UHPLC-MS/MS Analysis

Measurements were performed using an ultra-high-performance liquid chromatography–tandem mass spectrometry (UHPLC-MS/MS) system (Ultimate 3000RS coupled to a TSQ Quantis, Thermo Fisher Scientific, Waltham, MA, USA). Separation was performed on an Accucore C18 column (150 mm × 2.1 mm, 2.6 µm, Thermo Fisher Scientific, Waltham, MA, USA) equipped with an Accucore C18 guard column (10 mm × 2.1 mm, 2.6 µm) using gradient elution with 0.1% ammonia in water (solvent A) and 0.1% ammonia in methanol (solvent B). Solvent gradient was programmed as follows: 0.00 min, 30% B; 3.80 min, 100% B; 4.43 min, 100% B; 4.56 min, 30% B; and 7.59 min, 30% B. Total run time was 8 min. The flow rate was 0.474 mL/min. Column temperature was maintained at 30 °C, and the autosampler temperature at 10 °C. The needle was washed with a solvent mixture consisting of acetonitrile/methanol/water/formic acid = 40:40:20:1 *(v*/*v*/*v*/*v).* Sample injection volume was 1 µL. Heated electrospray ion source (H-ESI) was operated in positive ion mode with a spray voltage of 4800 V, while Sheath, Auxiliary, and Sweep gases were set to 45, 4.9, and 3.3 arbitrary units (Arb), respectively. The ion transfer tube temperature was set to 385 °C, and the vaporizer temperature was set to 400 °C. Collision-induced dissociation (CID) gas pressure was constant at 1.5 mTorr. Cycle time was 0.8 s, while both Q1 and Q3 quadrupoles were operated at unit resolution (0.7 FWHM). Scheduled multiple reaction monitoring (MRM) acquisition windows were applied. Transition details are summarized in Table 1.

Method validation was performed in accordance with ICH Q2(R2) and ICH M10 guidelines for the validation of analytical procedures. The range, number of concentration levels, and corresponding calibration curve equations are given in Supplementary Table S1 for each compound. All calibration curves showed good linearity with a correlation coefficient, R^2^ > 0.99. Limit of detection and limit of quantitation were determined based on a signal-to-noise ratio (S/N) of 3 for LOD and 10 for LOQ. Accuracy was calculated by comparing measured and nominal concentrations and expressed as a relative error (%RE). Precision was expressed as relative standard deviation (%RSD) for quality control samples (LLOQ, low, medium, and high QCs). Intra-day precision was measured by analysing five replicates of each QC prepared on the same day under the same conditions. Inter-day precision was measured by analysing QCs prepared over five consecutive days, while other parameters remained unchanged. The LLOQ corresponded to the lowest calibration level, while the LQC concentration was threefold higher than the LLOQ. The medium and high QC concentrations were 30–50% and above 75% of the calibration range. Robustness was tested by intentionally altering flow rate (±0.05 mL/min) and column temperature (±5 °C) and calculating %RSD. Specificity was ensured through the combination of retention times with qualifier and quantifier MRM transitions, as specified in Table 1. System suitability tests showed no carry-over and satisfactory repeatability for all analytes. All method validation results are presented in Supplementary Table S1.

Quantitative determination of nicotine, cotinine, and *trans*-3′-hydroxycotinine was performed using the peak area ratio of the analyte to the internal standard. Each calibration solution, prepared in a urine matrix, contained cotinine-*d3* at a concentration of 0.196 µg/mL. The internal standard was added to both calibration standards and urine samples prior to extraction as previously described. Calibration curves were constructed separately for high and low concentration ranges using unweighted linear regression and were forced through the origin (zero intercept). Data processing was performed using Chromeleon Chromatography Studio (version 7.2.10, Thermo Fisher Scientific, Waltham, MA, USA). Calibration curve equations, correlation coefficients (*R*^2^), and relative standard deviations (%RSD) are shown in the Supplementary Table S2.

### 2.8. Creatinine Determination

Creatinine measurements were performed with the enzymatic method using biochemical module c303 of the automatic analyser Cobas Pure, Roche Diagnostics GmbH (Mannheim, Germany). This enzymatic method is based on the conversion of creatinine by creatininase, creatinase, and sarcosine oxidase to glycine, formaldehyde and hydrogen peroxide. Catalysed by peroxidase, the liberated hydrogen peroxide reacts with 4-aminophenazone and HTIB (2,4,6-triodo-3-hydroxybenzoic acid) to form a quinone imine chromogen. The intensity of the colored quinoneimine dye is measured photometrically at 546 nm and is directly proportional to the creatinine concentration in the sample [27].

Urinary concentrations of nicotine, cotinine, and *trans*-3′-hydroxycotinine were normalized to creatinine levels to account for variations in urine dilution and expressed as ng/mg creatinine. The nicotine metabolite ratio (NMR) was calculated as the ratio of 3′-hydroxycotinine to cotinine [9].

### 2.9. Statistical Analysis

Descriptive statistics were used to summarize participants’ demographic characteristics, anthropometric measures, nicotine product use, and study outcomes. Because several continuous variables showed non-normal distributions, they are presented as median (interquartile range, IQR) together with minimum and maximum values, while categorical variables are presented as counts and percentages.

Repeated-measures ANOVA was used to assess changes in the analyzed continuous outcomes across study time points when the same participants were measured repeatedly. In additional analyses, mixed repeated-measures ANOVA was applied to examine whether these changes differed according to selected categorical variables, with time treated as the within-subject factor and the respective categorical variable as the between-subjects factor. Accordingly, the analyses evaluated the main effect of time, the main effect of group, and the time × group interaction. Before modelling, assumptions were assessed by inspecting boxplots for potential outliers, histograms and Q-Q plots for distributional shape, and the Shapiro–Wilk test for approximate normality. For models including a between-subjects categorical factor, homogeneity of variances across groups was assessed using Levene’s test, and homogeneity of covariance matrices was assessed using Box’s M test. The assumption of sphericity was tested using Mauchly’s test; when this assumption was violated, Greenhouse–Geisser-corrected results were interpreted. When appropriate, variables were natural log-transformed before inferential analyses to improve distributional properties and reduce the influence of extreme values. Estimated marginal means were used to describe factor-level patterns, and post hoc pairwise comparisons with appropriate Bonferroni-adjusted *p*-values for multiple testing were examined when indicated by significant overall effects.

Statistical significance was set at *p* < 0.05. Statistical analysis was performed using IBM SPSS Statistics, version 21 (IBM Corp., Armonk, NY, USA).

## 3. Results and Discussion

### 3.1. Participant Characteristics

Table 2 summarizes the demographic and nicotine use characteristics of the 72 participants included in the final analysis. As shown in Figure 1, four participants were excluded from the final analysis because of non-compliance with the study protocol.

Among the 26 users of alternative nicotine products, 17 used heated tobacco products, 8 used e-cigarettes, and 1 used nicotine pouches.

Information on lifestyle characteristics, including dietary habits, coffee consumption, and alcohol intake, was also collected. Most participants reported a balanced diet (55; 76.4%), while 11 (15.3%) preferred a high-protein diet, 5 (7.0%) reported a carbohydrate-rich diet, and 1 (1.3%) selected another dietary pattern. Most participants reported drinking 1–2 cups of coffee per day (56; 77.8%), whereas 11 (15.3%) reported higher coffee consumption. Alcohol intake was generally low, with 40 participants (55.6%) reporting rare alcohol consumption and 20 (27.8%) reporting 1–3 alcoholic drinks per week. No participant reported unusually high consumption of foods known to be rich in quercetin or other dietary factors potentially relevant to nicotine metabolism during the study period.

In the present study, lifestyle variables were not significantly associated with nicotine metabolism, as no significant differences were observed according to dietary habits, alcohol intake, or coffee consumption (all *p* > 0.05). Although previous studies have suggested that dietary constituents such as menthol, grapefruit, alcohol, and caffeic acid may influence CYP2A6 activity and nicotine metabolism [12,28], these effects were not observed in the present sample. None of the participants reported consuming menthol-containing products or grapefruit before urine sampling. Previous studies have also shown that alcohol consumption may influence nicotine metabolism, with higher alcohol intake being associated with an increased NMR [29], while caffeic acid has been reported to inhibit CYP2A6 activity, potentially slowing nicotine metabolism [24].

### 3.2. Urinary Nicotine and Nicotine Metabolite Concentrations

Urinary concentrations of nicotine, cotinine, 3-OH cotinine, and NMR across the four study time points are summarized in Table 3. Descriptively, median concentrations of nicotine, cotinine, 3-OH cotinine, and NMR were higher at the post-quercetin measurement than at baseline.

For log-transformed urinary nicotine, the assumption of sphericity was met (Mauchly’s W = 0.881, *p* = 0.116), and repeated-measures ANOVA showed no statistically significant overall effect of time (F(3, 213) = 2.494, *p* = 0.061, η_p_^2^ = 0.034). Bonferroni-adjusted pairwise comparisons indicated a significant difference between time points 1 and 3 (*p* = 0.021).

For log-transformed urinary cotinine, sphericity was met (Mauchly’s W = 0.888, *p* = 0.141), and no statistically significant overall time effect was observed (F(3, 213) = 2.314, *p* = 0.077, η_p_^2^ = 0.032). Although the linear within-subject contrast was significant (*p* = 0.025), Bonferroni-adjusted pairwise comparisons between time points were not statistically significant.

For log-transformed 3-OH cotinine, sphericity was met (W = 0.872, *p* = 0.088). Repeated-measures ANOVA showed a nominally significant effect of time (F(3, 213) = 2.699, *p* = 0.047, η_p_^2^ = 0.037), although the Greenhouse–Geisser-corrected result was no longer statistically significant (*p* = 0.052). The linear contrast was significant (F(1, 71) = 4.703, *p* = 0.033, η_p_^2^ = 0.062), whereas Bonferroni-adjusted pairwise comparisons between time points were not statistically significant.

For log-transformed NMR, sphericity was met (W = 0.940, *p* = 0.505), and no statistically significant effect of time was found (F(3, 213) = 0.382, *p* = 0.766, η_p_^2^ = 0.005). No significant within-subject contrasts or Bonferroni-adjusted pairwise differences were observed.

Previous studies have suggested that quercetin may influence nicotine metabolism [22,24], including possible modulation of CYP2A6-related pathways. Based on this rationale, the most relevant changes would be expected shortly after quercetin supplementation, particularly between the first and second measurements.

Although median nicotine concentrations were higher after quercetin supplementation, repeated-measures ANOVA showed no statistically significant overall time effect for log-transformed nicotine, cotinine, or NMR across the four study time points. Bonferroni-adjusted pairwise comparisons also showed no significant difference between the first and second measurements for log-transformed nicotine (*p* = 0.890), 3-OH cotinine (*p* = 0.292), or NMR (*p* = 1.000). Among the analyzed biomarkers, 3-OH cotinine showed the strongest temporal signal. However, this finding was weak, was not supported by Bonferroni-adjusted pairwise comparisons, and was attenuated after Greenhouse–Geisser correction.

### 3.3. Nicotine Metabolism Biomarkers by Sex

**Figure 2:**
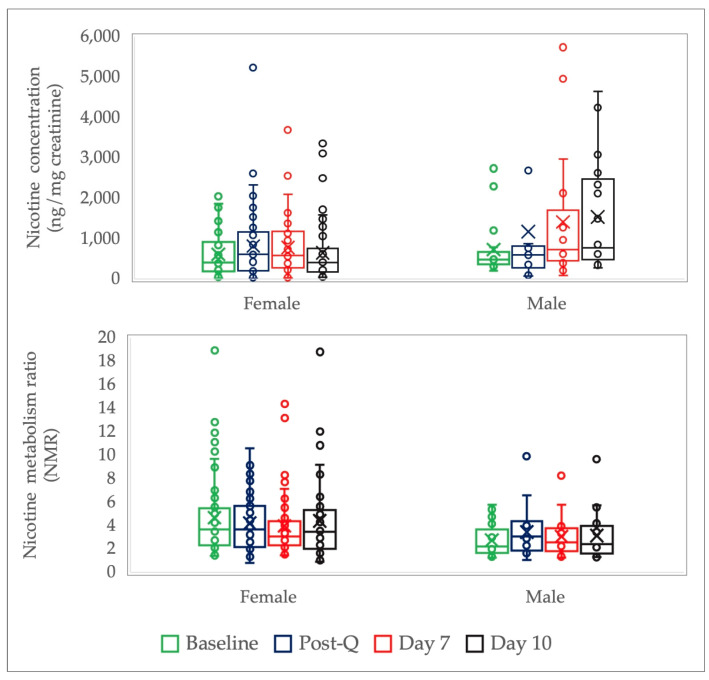
Comparison of urinary nicotine concentrations and nicotine metabolite ratio (NMR) across four time points, stratified by sex (female, n = 55; male, n = 17). Time points were defined as baseline (1), after 3 days of daily consumption of 500 mg quercetin (2), after 7 days of quercetin cessation (3), and after 10 days of quercetin cessation (4). Box plots show the median, interquartile range (box), and range (whiskers).

Nicotine concentrations and NMR values according to sex are presented in Figure 2 and Supplementary Table S3.

A repeated-measures ANOVA showed a significant effect of time on log-transformed urinary nicotine (F(3, 210) = 3.018, *p* = 0.031), a significant effect of sex (F(1, 70) = 4.299, *p* = 0.042), and a significant time-by-sex interaction (F(3, 210) = 3.312, *p* = 0.021), indicating that urinary nicotine changed differently over time in females and males. Post hoc analyses revealed a significant difference between sexes only at the fourth time point (*p* < 0.001), with higher urinary nicotine levels in males.

For log-transformed NMR, no significant effect of time or time-by-sex interaction was observed (*p* > 0.05). However, a significant main effect of sex was found (F(1, 70) = 4.183, *p* = 0.045), with females showing higher overall NMR values than males. A significant sex difference was observed only at the first time point (*p* = 0.013).

In the present study, sex-related differences were not uniform across all biomarkers. A sex-related pattern was observed for NMR, which was higher in females than in males, whereas urinary nicotine levels were higher in males at the fourth measurement.

Sex differences in nicotine metabolism have been reported previously, with women generally exhibiting faster nicotine metabolism, particularly during pregnancy and among users of oral contraceptives [5,30]. To reduce the influence of hormonal factors known to alter nicotine metabolism, pregnant women and women using oral contraceptives were excluded from the study, allowing a more controlled assessment of sex-related variation in the study sample.

### 3.4. Nicotine Metabolism Biomarkers by Type of Nicotine Product

The analysis included 46 participants who primarily used conventional cigarettes and 26 who primarily used alternative nicotine products. At baseline, urinary nicotine concentrations were similar between the two groups, as reflected by the absence of a significant between-group difference at the first measurement. Descriptive median values are presented in Supplementary Figure S1 and Table S4, with baseline medians of 438.5 ng/mg creatinine in conventional cigarette users and 475.1 ng/mg creatinine in users of alternative nicotine products. However, repeated-measures analyses showed that differences between groups were more evident in the temporal patterns of cotinine and 3-OH cotinine than in baseline nicotine levels alone.

Repeated-measures ANOVA showed significant time-by-group interactions for cotinine (F(3, 210) = 4.949, *p* = 0.002) and 3-OH cotinine (F(3, 210) = 3.095, *p* = 0.028), with higher levels in conventional cigarette users at time point 3. These findings suggest that downstream nicotine metabolites may be more sensitive to differences between conventional and alternative nicotine products than urinary nicotine or NMR.

Baseline urinary nicotine concentrations were comparable between users of conventional and alternative nicotine products, consistent with evidence indicating that nicotine exposure associated with some alternative nicotine products may be similar to, or slightly lower than, that observed with conventional cigarettes [20,31,32,33]. The observed differences in cotinine and 3-OH cotinine may reflect the considerable heterogeneity of alternative nicotine products, including differences in device characteristics, nicotine formulation, and patterns of use, all of which influence nicotine delivery [18,20,34].

Although some studies suggest that users of electronic nicotine delivery systems may be exposed to lower levels of certain harmful constituents than conventional cigarette smokers [35,36], comparable nicotine delivery does not imply that these products are risk-free. Both e-cigarettes and heated tobacco products have been associated with adverse respiratory and cardiovascular outcomes, while their long-term carcinogenic potential remains under investigation [15,16,37,38].

No significant effects of time, product group, or their interaction were observed for urinary nicotine or NMR, apart from a single between-group difference in nicotine at time point 3 (*p* = 0.032). Following quercetin supplementation, urinary nicotine showed only descriptive changes, while NMR remained stable. These findings suggest that grouping participants solely by product type may not fully capture variability in nicotine exposure or metabolism. Stratification by nicotine consumption intensity may therefore provide a more informative approach for evaluating biomarker changes following quercetin supplementation.

As noted in Section 3.1., the alternative nicotine product group included several product types. Because the number of participants in individual alternative product categories was small, separate analyses comparing e-cigarette, heated tobacco product, and nicotine pouch users were not performed. Therefore, the product-type results should be interpreted in relation to the overall comparison between conventional cigarette users and the combined alternative nicotine product group.

### 3.5. Nicotine Metabolism Biomarkers by Self-Reported Consumption

**Figure 3:**
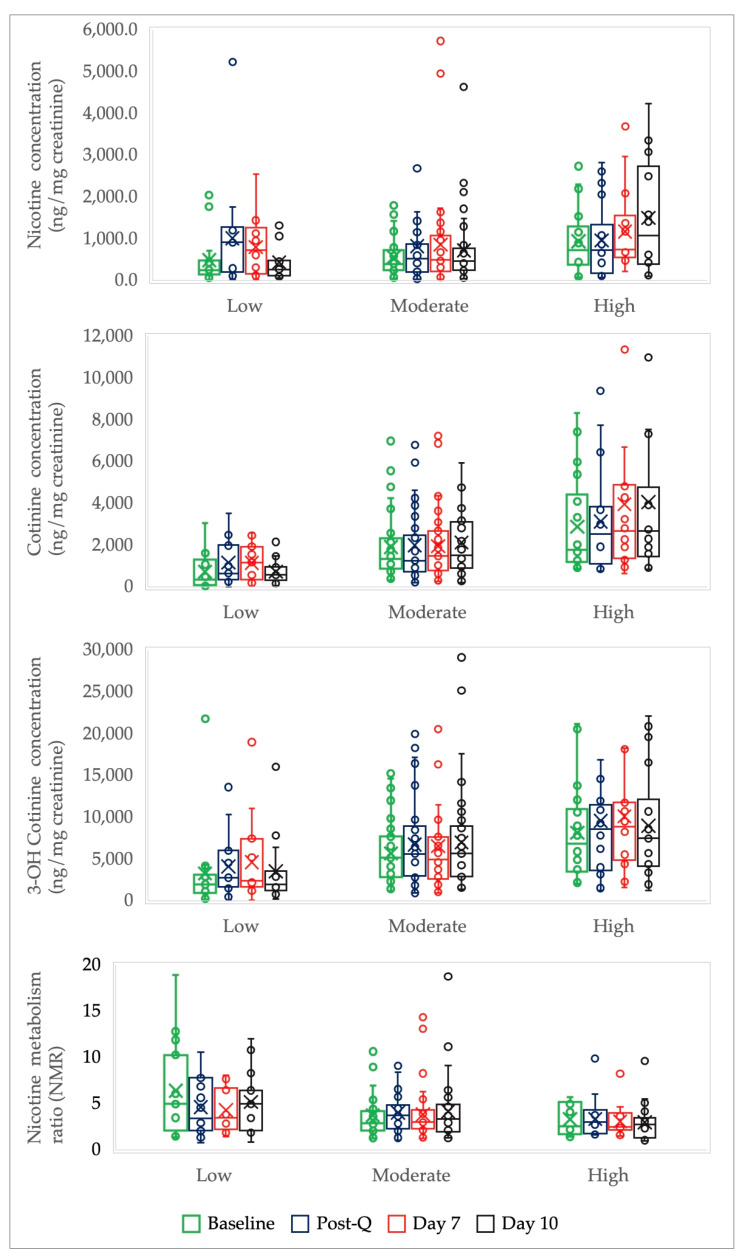
Comparison of urinary nicotine, cotinine, and 3-OH cotinine concentrations and the nicotine metabolite ratio (NMR) across four time points. Baseline (1), after 3 days of daily consumption of 500 mg of quercetin (2), after 7 days of cessation of quercetin consumption (3), and after 10 days of cessation of quercetin consumption (4), stratified according to self-reported consumption (low, moderate and high). Box plots show the median, interquartile range (box), and total (whiskers).

Nicotine, cotinine and 3-OH cotinine concentrations, as well as NMR values according to consumption category, are presented in Figure 3 and Supplementary Table S5.

As shown in Figure 3, cotinine and 3-OH cotinine levels were generally higher in the high consumption group than in the low consumption group, whereas differences in NMR across consumption groups were less pronounced.

Repeated-measures ANOVA showed a significant effect of time on urinary nicotine (F(3, 207) = 3.168, *p* = 0.025), but neither the time-by-consumption group interaction nor the between-subject effect of consumption category reached statistical significance (both *p* > 0.05). No significant effects of time, consumption group, or their interaction were observed for NMR.

In contrast, urinary cotinine showed a significant effect of time (F(3, 207) = 4.919, *p* = 0.003), a significant time-by-consumption group interaction (F(6, 207) = 3.181, *p* = 0.005), and a significant between-subject effect of consumption group (F(2, 69) = 14.285, *p* < 0.001). Similarly, 3-OH cotinine demonstrated a significant effect of time (F(3, 207) = 4.319, *p* = 0.006) and a significant between-subject effect of consumption group (F(2, 69) = 8.199, *p* < 0.001), whereas the interaction with time was not significant.

Temporal changes in cotinine and 3-OH cotinine were most evident in the low-consumption group, while moderate- and high-consumption groups showed relatively stable biomarker levels. Overall, cotinine and 3-OH cotinine concentrations were consistently higher in participants with greater nicotine consumption, suggesting that these downstream metabolites may be more sensitive indicators of short-term changes in nicotine exposure than urinary nicotine or NMR.

Previous research on quercetin and CYP2A6 has yielded inconsistent findings. Some studies have reported that quercetin inhibits CYP2A6 activity, whereas other evidence suggests that its effects may be context-dependent, possibly reflecting differences between in vitro and in vivo conditions and the influence of uncontrolled external factors [22,24]. CYP2A6 activity is known to be modulated by a variety of compounds, underscoring the complexity of nicotine metabolism and its susceptibility to external influences [39,40,41].

Nicotine metabolism is characterized by substantial interindividual variability resulting from a complex interplay of genetic, physiological, environmental, and behavioral factors. Factors such as CYP2A6 genetic variability, sex-related hormonal influences, dietary factors, medication use, and smoking-related factors may all contribute to differences in nicotine metabolism [5]. This variability may partly explain the absence of a consistent overall response to quercetin supplementation across the study population and the heterogeneous patterns observed in subgroup analyses. Given the complexity of nicotine metabolism and the multiple determinants of CYP2A6 activity described by Hukkanen et al. [5], longer intervention periods might be considered to detect lasting effects of quercetin supplementation. Although no consistent overall effect was observed, the exploratory subgroup patterns suggest that nicotine exposure characteristics may influence biomarker variability and should be considered in future studies of quercetin and nicotine metabolism.

### 3.6. Study Strengths, Limitations, and Future Directions

A strength of this study is that it examined multiple urinary biomarkers of nicotine metabolism across repeated measurements in participants with different levels of nicotine product consumption. To our knowledge, previous studies have not specifically examined short-term quercetin supplementation in relation to nicotine metabolism biomarkers across different levels of nicotine product consumption.

Several limitations should be considered when interpreting these findings. First, the absence of a placebo control group means that factors other than quercetin supplementation, such as day-to-day fluctuations in nicotine exposure or unmeasured behavioral changes, may have influenced the observed biomarker patterns. Although the within-subject design reduced interindividual variability by allowing each participant to serve as their own control, the results should be interpreted with appropriate caution.

The time since last nicotine product use before the first-morning urine sample was not recorded. This is particularly relevant for urinary nicotine, which has a short half-life and is strongly influenced by recent exposure. Therefore, urinary nicotine findings should be interpreted cautiously.

An additional limitation of the present study is that no formal a priori sample size calculation was performed. The observed overall effect sizes for changes over time were small, suggesting that the study had limited ability to detect subtle changes in urinary nicotine metabolism biomarkers. Therefore, the null overall findings should be interpreted as the absence of evidence for a robust short-term group-level effect, rather than definitive evidence that quercetin has no influence on nicotine metabolism. Future placebo-controlled studies should include formal sample size calculations based on a prespecified primary biomarker and a clinically meaningful effect size.

In addition, subgroup analyses according to sex, product type, and consumption intensity reduced the effective sample size within individual strata and therefore limited statistical power, especially for smaller groups. These analyses were exploratory and should not be interpreted as definitive evidence of subgroup-specific effects. The possibility that some observed subgroup differences occurred by chance cannot be excluded. Future studies should include larger and more balanced samples with prespecified subgroup hypotheses.

Although this study provides useful insight into the relationship between quercetin supplementation and nicotine metabolism across different levels of nicotine exposure, nicotine metabolism is a complex process influenced by multiple genetic, environmental, and behavioral factors that cannot be fully controlled under real-life conditions. Furthermore, the present findings are limited to biochemical markers of nicotine metabolism, and their potential implications for nicotine dependence, withdrawal, or smoking cessation outcomes remain unclear and require further investigation.

The study also relied on self-reported data regarding nicotine product use and related behaviors, which may have introduced reporting bias. Similarly, adherence to quercetin supplementation was assessed through participant self-report and was not verified using objective measures. Therefore, some degree of misclassification of compliance cannot be completely excluded. In addition, despite the use of structured questionnaires, certain aspects of product variability, including differences in device characteristics, nicotine concentrations, and patterns of use, could not be fully standardized. Future research should further clarify these findings in real-world settings, include longer supplementation periods, employ randomized placebo-controlled designs, and consider repeated supplementation cycles to better assess the longer-term effects of quercetin on nicotine metabolism.

## 4. Conclusions

Short-term quercetin supplementation was not associated with a consistent overall change in urinary nicotine metabolism biomarkers in the total sample. Among the analyzed biomarkers, *trans*-3′-hydroxycotinine showed the strongest temporal signal; however, this finding was weak and should be interpreted as exploratory. Subgroup analyses indicated that biomarker patterns varied according to sex, nicotine product type, and particularly nicotine consumption intensity, with cotinine and *trans*-3′-hydroxycotinine showing clearer temporal and between-group patterns than the nicotine metabolite ratio. However, these subgroup findings were not uniform across biomarkers and may have been influenced by reduced statistical power within individual strata. Overall, the present findings do not provide definitive evidence of a robust short-term effect of quercetin on nicotine metabolism, but suggest that downstream nicotine metabolites and individual exposure characteristics warrant further investigation. Larger randomized placebo-controlled studies with prespecified endpoints, formal sample size calculations, longer supplementation periods, and more frequent sampling are needed to confirm these observations and determine their biological and clinical relevance.

## Figures and Tables

**Table 1:** Compound transition details.

Compound	RT (min)	Start (min)	End (min)	Q1 (*m*/*z*)	Q3 ^a^ (*m*/*z*)	CE ^a^ (V)	Q3 ^b^ (*m*/*z*)	CE ^b^ (V)	RF (V)
nicotine	4.75	3.5	6.0	163	130	19	117	24	66
cotinine	1.70	0.5	3.0	177	80	23	98	19	86
*trans*-3′-hydroxycotinine	1.30	0	2.5	193	80	27	134	17	90
cotinine-*d3*	1.70	0.5	3.0	180	80	24	101	21	104

Abbreviations: RT—retention time; Q1—precursor ion; Q3—product ion (^a^—quantifier, ^b^—qualifier); CE—collision energy; RF—focusing lens voltage.

**Table 2:** Participant characteristics and nicotine use patterns.

	n (%)	Min–Max
Age; median (IQR)	45.00 (20.00)	19.00–74.00
BMI; median (IQR)	24.36 (5.31)	18.42–33.95
Sex; n (%)		
Female	55 (76.4)	
Male	17 (23.6)	
Type of nicotine product consumed; n (%)		
Conventional cigarettes	46 (63.9)	-
Alternative nicotine products	26 (36.1)	-
Reported consumption; n (%)		
Low	15 (20.8)	-
Moderate	39 (54.2)	-
High	18 (25.0)	-

BMI, body mass index; IQR, interquartile range. Alternative nicotine products included e-cigarettes, heated tobacco products, and nicotine pouches. Consumption categories (low, moderate, and high) are defined in Section 2.4.

**Table 3:** Minimum, maximum, median, and interquartile range of urinary nicotine, cotinine, 3-OH cotinine, and nicotine metabolite ratio (NMR) across four study time points: baseline, post-quercetin, Day 7, and Day 10 (n = 72).

		Median	IQR	Minimum	Maximum
Nicotine (ng/mg creatinine)	Baseline	443.39	625.10	18.59	2736.98
	Post-Q	603.16	876.57	9.14	8015.91
	Day 7	630.02	870.53	28.98	5735.94
	Day 10	474.38	968.05	48.99	4638.47
Cotinine (ng/mg creatinine)	Baseline	1311.46	1362.45	21.38	8318.47
	Post-Q	1377.07	2105.23	13.07	9391.16
	Day 7	1564.78	1848.22	18.89	15,648.13
	Day 10	1501.75	2198.76	55.39	17,258.46
3-OH cotinine (ng/mg creatinine)	Baseline	4252.83	5393.10	118.89	21,823.47
	Post-Q	5399.50	6588.63	73.92	40,493.08
	Day 7	5383.16	6222.70	58.15	36,189.41
	Day 10	4672.60	6450.61	192.81	29,114.20
NMR	Baseline	3.12	3.12	1.26	18.92
	Post-Q	3.36	2.73	0.82	10.59
	Day 7	3.04	2.00	1.25	14.35
	Day 10	3.26	3.12	0.89	18.79

Baseline, before quercetin supplementation; Post-Q, after 3 days of daily quercetin supplementation (500 mg/day); Day 7, after 7 days of quercetin cessation; Day 10, after 10 days of quercetin cessation. NMR, nicotine metabolite ratio; IQR, interquartile range.

## Data Availability

The raw data supporting the conclusions of this article will be made available by the authors on request.

## Supplementary Materials

The following supporting information can be downloaded at https://www.mdpi.com/article/10.3390/toxics14070591/s1. Table S1: Method validation results for nicotine, cotinine, and *trans*-3′-hydroxycotinine; Table S2: Analytical method data for nicotine, cotinine, and *trans*-3′-hydroxycotinine; Table S3: Median, interquartile range, minimum, and maximum values of urinary nicotine metabolism biomarkers across four study time points, stratified by sex (female, n = 55; male, n = 17); Table S4: Median, interquartile range, minimum, and maximum values of urinary nicotine metabolism biomarkers across four study time points, stratified by type of nicotine product (conventional cigarettes, n = 46; alternative nicotine products, n = 26); Table S5: Median, interquartile range, minimum, and maximum values of urinary nicotine metabolism biomarkers across four study time points by consumption category (low, n = 15; moderate, n = 39; high, n = 18; total n = 72); Figure S1: Comparison of urinary nicotine, cotinine, and 3-OH cotinine concentrations and the nicotine metabolite ratio (NMR) across four time points, stratified by type of nicotine products used (conventional cigarettes, n = 46; alternative nicotine products, n = 26).
