# Supplementary material for: Effects of Short-Term Quercetin Supplementation on Urinary Nicotine Metabolism Biomarkers in Users of Conventional and Alternative Nicotine Products: A Repeated-Measures Study
Source: Toxics. 2026 Jul 5;14(7):591. doi: 10.3390/toxics14070591 (PMC13431417; doi:10.3390/toxics14070591)
Supplement: Supplementary file 1 [file toxics-14-00591-s001.zip › toxics-4402439-supplementary.pdf]

Table S1. Method validation results for nicotine, cotinine, and *trans*-3′-hydroxycotinine.

	**nicotine**	**cotinine**	***trans*-3′-hydroxycotinine**
**Range** (µg/mL)	0.05–1.0	0.10–2.0	0.25–5.0
**Concentration levels**	5	6	6
**Calibration curve equation**	y = 9696x+14	y = 11574x−1115	y = 9106x−1373
**Linearity** (R^2)	0.9987	0.9930	0.9996
**LOD** (µg/mL)	0.005	0.004	0.021
**LOQ** (µg/mL)	0.016	0.012	0.063
**LLOQ** (µg/mL)	0.05	0.10	0.25
**LQC** (µg/mL)	0.15	0.30	0.75
**MQC** (µg/mL)	0.40	0.80	2.0
**HQC** (µg/mL)	0.80	1.6	4.0
**Accuracy** (%RE)	1.44–5.48	4.03–10.12	0.08–6.62
**Intra-day precision** (%RSD)	3.03–8.12	0.41–4.90	2.57–15.33
**Inter-day precision** (%RSD)	11.10–16.56	5.96–14.02	12.40–18.09
**Robustness** – flow rate	RSD < 24.37%	RSD < 4.52%	RSD < 9.03%
**Robustness** – column temperature	RSD < 12.85%	RSD < 10.03%	RSD < 21.13%
**Specificity**	technology inherent justification		
**System suitability**	no carry-over		
**System repeatability** (%RSD)	3.08	0.71	1.48

Abbreviations: R2 – correlation coefficient; LOD – limit of detection; LOQ – limit of quantitation; LLOQ – low limit of quantification; LQC – low quality control, MQC – medium quality control; HQC – high quality control; RE – relative error; RSD – relative standard deviation

Table S2. Analytical method data for nicotine, cotinine, and *trans*-3′-hydroxycotinine

a) high concentration range

**Compound**	**Range** (µg/mL)	***n***	**Calibration curve equation**	R^2	**RSD** (%)
nicotine	0.1 – 10.0	5	y = 8997x	0.997	6.75
cotinine	0.1 – 20.0	6	y = 2621x	0.992	10.05
trans-3-hydroxycotinine	1.0 – 100	6	y = 686x	0.999	3.44

b) low concentration range

**Compound**	**Range** (µg/mL)	***n***	**Calibration curve equation**	R^2	**RSD** (%)
nicotine	0.001 – 1.0	6	y = 5833x	0.995	8.37
cotinine	0.01 – 2.0	5	y = 2953x	0.999	4.07
trans-3-hydroxycotinine	0.005 – 5.0	6	y = 571x	0.999	4.87

Abbreviations: *n* – number of points; R^2 – correlation coefficient; RSD – relative standard deviation

Table S3. Median, interquartile range, minimum, and maximum values of urinary nicotine metabolism biomarkers across four study time points, stratified by sex. (female, n = 55; male, n = 17).

	Time point	Sex	Median	IQR	Minimum	Maximum
Nicotine ng/mg creatinine	Baseline	Female	410.37	711.93	18.59	2199.33
		Male	477.70	181.90	203.93	2736.98
	Post-Q	Female	609.85	921.300	9.14	5238.62
		Male	596.46	403.16	77.75	8015.91
	Day 7	Female	577.69	885.17	28.98	3688.77
		Male	727.12	772.23	88.33	5735.94
	Day 10	Female	412.62	497.69	48.99	3356.37
		Male	779.08	1842.69	274.97	4638.47
Cotinine ng/mg creatinine	Baseline	Female	1222.49	1455.00	21.38	8318.47
		Male	1847.34	957.19	859.51	4791.38
	Post-Q	Female	1207.14	1677.54	13.07	9391.16
		Male	2051.60	2982.16	205.96	7740.37
	Day 7	Female	1502.31	1826.14	18.89	15648.13
		Male	2270.22	2182.93	275.98	7240.01
	Day 10	Female	1143.59	2247.35	55.39	17258.46
		Male	2210.82	1783.63	782.51	10982.78
3-OH cotinine ng/mg creatinine	Baseline	Female	4227.12	5602.22	118.89	21823.28
		Male	4435.03	4109.36	1178.50	13790.85
	Post-Q	Female	5177.81	5923.70	73.92	40493.08
		Male	5621.19	8354.89	919.66	17029.82
	Day 7	Female	5226.29	5762.33	58.15	36189.41
		Male	5990.04	6109.28	925.90	16479.45
	Day 10	Female	4255.78	6405.77	192.81	29114.20
		Male	6774.88	5412.51	1405.29	19619.32
NMR	Baseline	Female	3.64	3.06	1.38	18.92
		Male	2.19	1.56	1.26	5.74
	Post-Q	Female	3.68	3.16	0.82	10.59
		Male	3.05	2.44	1.03	9.91
	Day 7	Female	3.08	2.02	1.47	14.35
		Male	2.57	1.74	1.25	8.24
	Day 10	Female	3.47	3.22	0.89	18.79
		Male	2.40	2.05	1.30	9.63

Abbreviations: Baseline, before quercetin supplementation; Post-Q, after 3 days of daily quercetin supplementation (500 mg/day); Day 7, after 7 days of quercetin cessation; Day 10, after 10 days of quercetin cessation. NMR, nicotine metabolite ratio; IQR, interquartile range.

Table S4. Median, interquartile range, minimum, and maximum values of urinary nicotine metabolism biomarkers across four study time points, stratified by type of nicotine product. (conventional cigarettes, n = 46; alternative nicotine products, n = 26).

	Time point	Types of nicotinic preparations	Median	IQR	Minimum	Maximum
Nicotine ng/mg creatinine	Baseline	Conventional cigarettes	438.50	698.47	65.41	2736.98
		Alternative nicotine products	475.07	557.19	18.59	1868.04
	Post-Q	Conventional cigarettes	699.96	873.72	37.09	8015.91
		Alternative nicotine products	357.99	538.37	9.14	5238.62
	Day 7	Conventional cigarettes	677.85	872.59	89.23	5735.94
		Alternative nicotine products	488.31	920.91	28.98	3688.77
	Day 10	Conventional cigarettes	483.66	1046.04	54.04	4638.47
		Alternative nicotine products	468.68	556.92	48.99	1719.27
Cotinine ng/mg creatinine	Baseline	Conventional cigarettes	1311.46	1486.94	79.84	7428.21
		Alternative nicotine products	1332.60	1315.34	21.38	8318.47
	Post-Q	Conventional cigarettes	1284.33	2472.00	213.67	9391.16
		Alternative nicotine products	1652.65	1625.47	13.07	6802.91
	Day 7	Conventional cigarettes	1792.46	2162.52	292.98	15648.13
		Alternative nicotine products	1271.94	1613.14	18.89	6702.67
	Day 10	Conventional cigarettes	1501.75	2088.55	156.82	17258.46
		Alternative nicotine products	1333.60	2212.93	55.39	5036.83
3-OH cotinine ng/mg creatinine	Baseline	Conventional cigarettes	4288.85	4942.21	260.13	21823.28
		Alternative nicotine products	4154.19	5700.52	118.89	15275.97
	Post-Q	Conventional cigarettes	6171.86	7519.35	492.96	40493.08
		Alternative nicotine products	4640.47	3919.50	73.92	17175.28
	Day 7	Conventional cigarettes	6013.29	7509.64	1258.37	36189.41
		Alternative nicotine products	4166.28	4810.99	58.15	18311.90
	Day 10	Conventional cigarettes	6164.90	6451.14	787.65	25178.99
		Alternative nicotine products	3835.83	5462.26	192.81	29114.20
NMR	Baseline	Conventional cigarettes	3.35	3.16	1.32	12.82
		Alternative nicotine products	2.81	2.89	1.26	18.92
	Post-Q	Conventional cigarettes	3.54	2.60	0.82	10.59
		Alternative nicotine products	3.28	2.61	1.32	8.41
	Day 7	Conventional cigarettes	2.73	1.67	1.25	14.35
		Alternative nicotine products	3.51	1.69	1.50	8.29
	Day 10	Conventional cigarettes	3.04	3.30	0.89	12.01
		Alternative nicotine products	3.38	2.18	1.06	18.79

Abbreviations: Baseline, before quercetin supplementation; Post-Q, after 3 days of daily quercetin supplementation (500 mg/day); Day 7, after 7 days of quercetin cessation; Day 10, after 10 days of quercetin cessation. NMR, nicotine metabolite ratio; IQR, interquartile range.

Table S5. Median, interquartile range, minimum, and maximum values of urinary nicotine metabolism biomarkers across four study time points by consumption category (low, n = 15; moderate, n = 39; high, n = 18; total n = 72).

	Time point	Consumption category	Median	IQR	Minimum	Maximum
Nicotine (ng/mg creatinine)	Baseline	Low	252.17	331.29	18.59	2047.85
		Moderate	397.27	491.84	50.45	1868.04
		High	726.35	909.04	78.80	2736.98
	Post-Q	Low	913.27	1075.44	9.14	5238.62
		Moderate	519.01	670.39	37.09	8015.91
		High	725.84	1162.95	80.27	2828.43
	Day 7	Low	736.18	1108.23	28.98	2552.46
		Moderate	498.46	859.92	35.63	5735.94
		High	750.04	1005.59	216.06	3688.77
	Day 10	Low	266.41	357.33	54.04	1324.11
		Moderate	473.20	533.50	48.99	4638.47
		High	1083.55	2350.66	107.73	4242.80
NMR	Baseline	Low	5.03	8.21	1.38	18.92
		Moderate	2.90	2.14	1.26	11.11
		High	2.55	3.47	1.45	5.74
	Post-Q	Low	3.40	5.72	0.82	10.59
		Moderate	3.74	2.59	1.03	9.65
		High	3.02	2.59	1.64	9.91
	Day 7	Low	3.49	4.48	1.47	8.11
		Moderate	3.05	2.06	1.25	14.35
		High	2.51	1.87	1.50	8.24
	Day 10	Low	5.02	4.35	0.89	12.01
		Moderate	3.33	2.94	1.30	18.79
		High	2.75	2.19	1.02	9.63
Cotinine (ng/mg creatinine)	Baseline	Low	339.27	1220.14	21.38	3045.37
		Moderate	1332.23	1450.23	332.33	6987.12
		High	1788.11	3214.82	874.71	8318.47
	Post-Q	Low	651.33	1675.26	13.07	3539.38
		Moderate	1263.32	1736.54	205.96	6802.91
		High	2542.64	2722.98	761.14	9391.16
	Day 7	Low	1172.50	1590.95	18.89	2600.53
		Moderate	1495.01	1879.63	275.98	7240.01
		High	2669.13	3523.76	631.20	15648.13
	Day 10	Low	593.09	639.24	55.39	2158.65
		Moderate	1523.61	2213.10	215.54	5939.03
		High	2679.82	3316.01	843.52	17258.46
3-OH cotinine (ng/mg creatinine)	Baseline	Low	1966.48	2246.65	118.89	21823.28
		Moderate	5177.95	4870.95	1178.50	15275.97
		High	6869.36	7445.19	2027.11	21181.47
	Post-Q	Low	2776.90	4389.16	73.92	13625.52
		Moderate	5621.19	6014.91	919.66	19999.81
		High	8577.24	7837.07	1272.54	40493.08

	Day 7	Low	2426.54	5787.52	58.15	18998.10
		Moderate	4958.54	5039.31	925.90	36017.12
		High	8917.26	6883.00	1638.98	36189.41
	Day 10	Low	2007.43	2340.44	192.81	16030.27
		Moderate	5672.82	6061.13	1405.29	29114.20
		High	7528.90	8011.11	1207.88	22166.82

Abbreviations: Baseline, before quercetin supplementation; Post-Q, after 3 days of daily quercetin supplementation (500 mg/day); Day 7, after 7 days of quercetin cessation; Day 10, after 10 days of quercetin cessation. NMR, nicotine metabolite ratio; IQR, interquartile range.

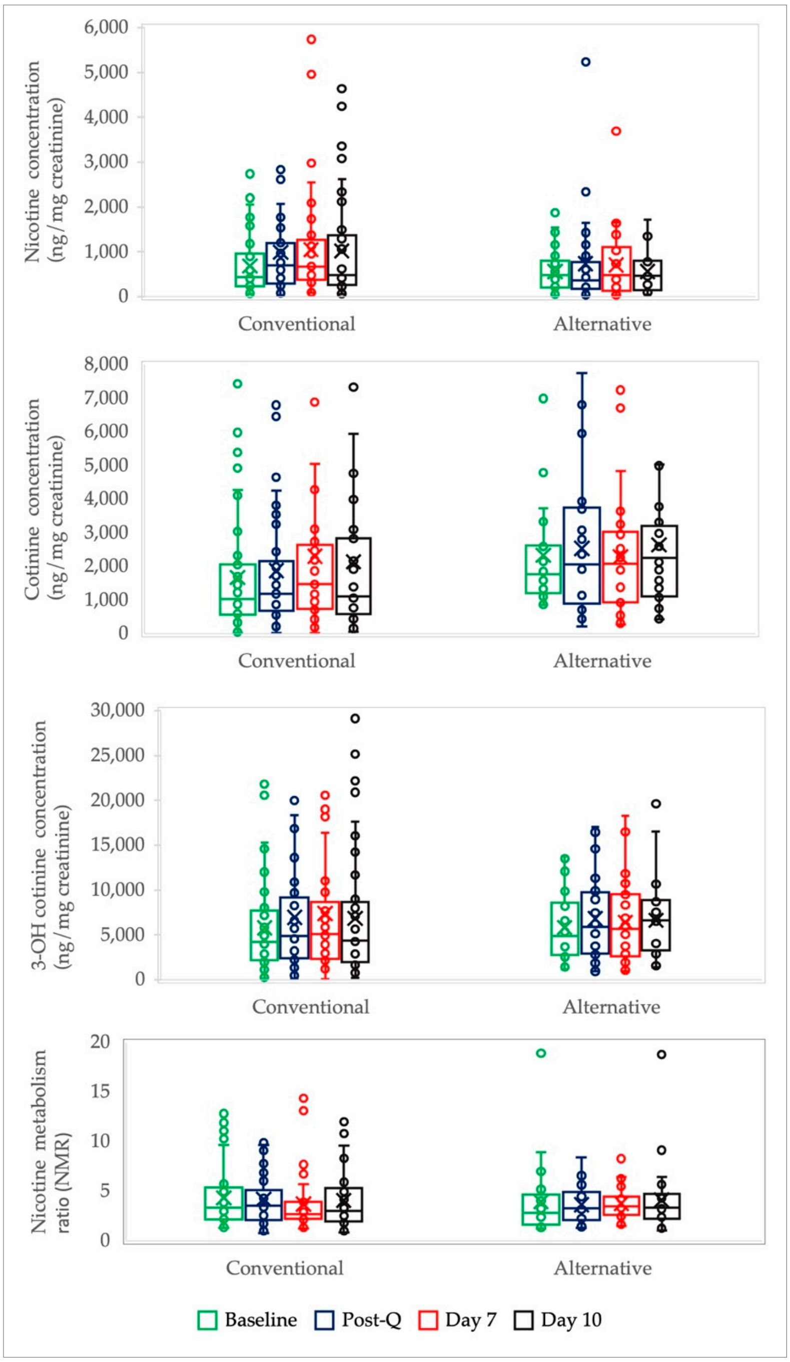

Figure S1. Comparison of urinary nicotine, cotinine, and 3-OH cotinine concentrations and the nicotine metabolite ratio (NMR) across four time points, stratified by type of nicotine products used (conventional cigarettes, n = 46; alternative nicotine products, n = 26). Time points were defined as baseline (1), after 3 days of daily consumption of 500 mg quercetin (2), after 7 days of quercetin cessation (3), and after 10 days of quercetin cessation (4). Box plots show the median, interquartile range (box), and range (whiskers).
